# Chemotherapeutic Agent Paclitaxel Mediates Priming of NLRP3 Inflammasome Activation

**DOI:** 10.3389/fimmu.2019.01108

**Published:** 2019-05-16

**Authors:** Seunghwan Son, Do-Wan Shim, Inhwa Hwang, Jong-Hwan Park, Je-Wook Yu

**Affiliations:** ^1^Department of Microbiology and Immunology, Institute for Immunology and Immunological Diseases, Brain Korea 21 PLUS Project for Medical Science, Yonsei University College of Medicine, Seoul, South Korea; ^2^BK 21 PLUS Project Team, Laboratory Animal Medicine, College of Veterinary Medicine, Chonnam National University, Gwangju, South Korea

**Keywords:** paclitaxel, chemotherapy, NLRP3, inflammasome, caspase-1, Toll-like receptor 4, interleukin-1beta

## Abstract

Paclitaxel is a chemotherapeutic drug commonly used to treat different types of cancer. In addition to its antitumor effect, paclitaxel is also known to promote Toll-like receptor (TLR) 4-dependent inflammatory responses, which may lower its chemotherapeutic efficacy. However, it remains unclear whether paclitaxel is able to affect inflammasome signaling in myeloid or cancer cells. Therefore, we examined the potential effect of paclitaxel on the activation of an inflammasome complex by examining caspase-1 activation and interleukin (IL)-1β secretion in bone marrow-derived macrophages (BMDMs). The results showed that treatment with paclitaxel alone or following LPS priming failed to trigger the secretion of active caspase-1 and IL-1β from BMDMs. However, paclitaxel could induce robust activation of caspase-1 in BMDMs in the presence of NLRP3 inflammasome-activating signal 2, such as ATP or nigericin. This paclitaxel/ATP-mediated inflammasome activation was completely abrogated in *Nlrp3*-deficient macrophages. Mechanistically, paclitaxel treatment induced robust activation of the TLR4 signaling cascade, including phosphorylation of IκB and JNK and upregulation of proinflammatory cytokine mRNA levels in a TLR4-dependent manner. In contrast, paclitaxel treatment alone did not induce mitochondrial damages such as the loss of the mitochondrial membrane potential and production of mitochondrial ROS. These findings suggest that paclitaxel can drive the priming of signal-mediated events for NLRP3 activation but not a second signal-triggered phenomenon such as mitochondrial damage. This suggestion was supported by the observations that paclitaxel treatment caused robust IL-1β production in macrophages in the presence of cell-free medium derived from growth of injured cells and also in the spleen of mice. Collectively, our data strongly indicate that paclitaxel is able to facilitate the activation of NLRP3 inflammasome signaling in a certain physiological environment.

## Introduction

Paclitaxel is a common chemotherapeutic drug used for treating a wide range of solid tumors, including lung, ovarian, and breast cancers (1). Although the precise mechanism of its cytotoxicity is still debatable, microtubule stabilization is considered the main mode of action of paclitaxel (1, 2). Paclitaxel-stabilized microtubules may interfere with normal mitosis, eventually leading to apoptotic cell death (2).

In addition to its chemotherapeutic effect, paclitaxel has been demonstrated to induce inflammation in myeloid or cancer cells and in the paclitaxel-treated patients (3–6). In particular, paclitaxel is known to trigger the production of proinflammatory cytokines such as interleukin (IL)-6 and IL-8 via binding to Toll-like receptor 4 (TLR4) on myeloid cells and many cancer cells (7). Of note, paclitaxel-induced inflammation often contributes to tumor proliferation, invasion, and chemoresistance during paclitaxel treatment (7–9). Meanwhile, a recent study has proposed that paclitaxel reprograms M2-polarized tumor-associated macrophages to M1-like phenotype in a TLR4-dependent manner, and this M1-like phenotype contributes to the antitumor effect of paclitaxel (10). Hence, the molecular mechanism of paclitaxel-induced inflammation needs to be further elucidated to understand the efficacy of paclitaxel chemotherapy.

The TLR4-NF-κB signaling axis is considered the main pathway for paclitaxel-induced production of proinflammatory cytokines (7). Most proinflammatory cytokines are released from myeloid and non-myeloid cells upon activation of TLR4-NF-κB pathways. However, active IL-1β, a key proinflammatory cytokine, can only be secreted upon the formation of an intracellular inflammasome complex (11, 12). Interestingly, IL-1β may promote tumorigenesis and tumor invasion (13), and the blockade of the IL-1 receptor by an IL-1R antagonist was shown to improve the anti-tumor effect of chemotherapy (14, 15). Therefore, it seems intriguing to examine whether paclitaxel treatment facilitates the assembly and activation of an inflammasome complex and subsequent secretion of IL-1β.

The inflammasome complex comprises of sensor molecules such as NOD-like receptor (NLR) family, pyrin domain-containing 3 (NLRP3) or NLR family, card domain-containing 4 (NLRC4), apoptosis-associated speck-like protein containing a caspase recruitment domain (ASC), and procaspase-1 (12). In a resting state, sensor molecules are thought to be inactive and thus incapable of binding to the ASC protein (16). Upon stimulation with inflammasome-activating signals, sensor molecules may be converted into an active form and assemble an inflammasome complex, which leads to the activation of caspase-1. Subsequently, active caspase-1 processes inactive pro-IL-1β into an active mature form (17). Some previous reports have demonstrated that paclitaxel treatment led to the increased IL-1β mRNA production and protein secretion (18, 19). However, the inflammasome/caspase-1-activating capacity of paclitaxel has not been carefully studied yet. Here, we examined whether paclitaxel is able to drive the assembly of the inflammasome complex and activation of caspase-1 in macrophages.

## Materials and Methods

### Reagents and Antibodies

Paclitaxel, doxorubicin, etoposide, LPS, ATP, nigericin, staurosporine, glibenclamide, and valiomycin were purchased from Sigma-Aldrich. Ciliobrevin D and SP600125 were purchased from Calbiochem. JC-1 and MitoSOX were purchased from Invitrogen. Alum was purchased from InvivoGen. Anti-mouse caspase-1, anti-NLRP3 and anti-ASC antibodies were obtained from AdipoGen. Anti-mouse IL-1β antibody was obtained from R&D Systems. Anti-phospho-IκB and anti- IκB antibodies were purchased from Cell Signaling Technology. Anti-phospho-JNK and anti-JNK antibodies were obtained from Invitrogen and BD, respectively. Anti-β-actin antibody was purchased from Santa Cruz Biotechnology.

### Mice and Cell Cultures

C57BL/6, *Nlrp3*^−/−^, and *Tlr4*^−/−^ mice were obtained from The Jackson Laboratory. All mice were maintained under specific pathogen-free conditions and 9~15-weeks-old male mice were used for experiments. In some experiments, mice were intraperitoneally injected with PBS or paclitaxel (40 mg/kg) and sacrificed 24 h post-injection. Mouse bone marrow cells were prepared from the femurs of C57BL/6, *Nlrp3*^−/−^, and *Tlr4*^−/−^ mice and cultured in DMEM supplemented with L929 culture supernatants for 5~7 days to differentiate into bone marrow-derived macrophages (BMDMs). Protocols for the animal experiments (2017-0221) were approved by the Institutional Ethical Committee, Yonsei University College of Medicine. All experiments were performed in accordance with the approved guidelines of the Institutional Ethical Committee. BMDMs were maintained in L929-conditioned DMEM supplemented with 10% FBS and antibiotics. A549 cells were grown in DMEM supplemented with 10% FBS and antibiotics.

### Immunoblot Analysis

Cells were lysed in buffer containing 20 mM HEPES (pH 7.5), 0.5% Nonidet P-40, 50 mM KCl, 150 mM NaCl, 1.5 mM MgCl_2_, 1 mM EGTA, and protease inhibitors. Soluble lysates were fractionated by SDS-PAGE, and separated proteins were transferred to PVDF membranes. In some experiments, cell culture supernatants were precipitated by methanol/chloroform as described previously (20) and then immunoblotted. All the blots shown are representative images from at least three-independent experiments.

### mRNA Quantification

To measure mRNA expression, total RNA was isolated from cells using a TRIzol reagent (Invitrogen) and reverse transcribed using PrimeScript RT Master Mix (Takara). Quantitative PCR was performed using SYBR Premix Ex Taq (Takara) and the following primers: 5′-GCC CAT CCT CTG TGA CTC AT-3′ and 5′-AGG CCA CAG GTA TTT TGT CG-3′ (mouse *Il-1*β); 5′-AGT TGC CTT CTT GGG ACT GA-3′ and 5′-TCC ACG ATT TCC CAG AGA AC-3′ (mouse *Il-6*); 5′- ATG CTG CTTCGA CAT CTC CT-3′ and 5′-AAC CAA TGC GAG ATC CTG AC-3′ (mouse *Nlrp3*); and 5′-CGC GGT TCT ATT TTG TTG GT-3′ and 5′-AGT CGG CAT CGT TTA TGG TC-3′ (mouse *Rn18s*).

### Assay of Inflammasome Activation and Cell Death

To stimulate the conventional NLRP3 inflammasome activation, BMDMs were primed with LPS (0.25 μg/mL, 3 h), followed by treatment with ATP (2.5 mM, 30 min), nigericin (5 μM, 40 min) or alum (250 μg/mL, 6 h). Inflammasome activation was determined by the presence of protein bands corresponding to active caspase-1 p20 and active IL-1β band in immunoblots of culture supernatants. To measure extracellular levels of IL-1β or IL-6, culture supernatants were assayed using an IL-1β- or IL-6-specific ELISA kit (BioLegend) according to the manufacturer's instructions. Caspase-1-dependent cell death was determined by extracellular release of lactate dehydrogenase (LDH) using a CytoTox96 non-radioactive cytotoxicity assay kit (Promega). The LDH release was calculated as [extracellular LDH/(intracellular LDH + extracellular LDH) × 100].

### Assay of Inflammasome Assembly

To determine the oligomerization of ASC, a discuccinimidyl suberate (DSS; Thermo Scientific)-mediated cross-linking assay was performed as described previously (21). To visualize molecular interactions of NLRP3 with ASC, a proximity-ligation (PL) assay was performed using the Duolink *in situ* Red starter kit (Sigma) with an anti-ASC and anti-NLRP3 antibodies according to the manufacturer's protocols. The relative number of PL signal-positive cells was quantified using the Image J software.

### Measurement of Mitochondrial Membrane Potential and Mitochondrial ROS Production

To measure the mitochondrial membrane potential, cells were stained with the membrane potential-sensitive JC-1 dye, which forms red fluorescence-emitting aggregates on polarized mitochondria and green fluorescence-emitting monomers on depolarized mitochondria. Cells were analyzed by flow cytometry using FL1 and FL2 channels. To measure mitochondrial ROS production levels, mouse BMDMs were stained with MitoSOX (Invitrogen) after appropriate treatments. Cells were then analyzed by flow cytometry (FACSVerse, BD) based on the level of MitoSOX.

### Cultures in Conditioned Medium

To examine the effects of damaged cell-derived factors, A549 cells were first treated with staurosporine (1 μg/mL) for 24 h, then washed with PBS, and incubated with fresh Opti-MEM for an additional 18 h. The cell-free culture medium was then collected from the A549 cells and mixed with BMDM culture medium (2:1 ratio). This conditioned medium was added to BMDMs, and its effects were assayed in appropriate experiments.

### Statistical Analysis

All values were expressed as the mean ± SEM of individual samples. Data were analyzed using one-way analysis of variance followed by Dunnett's *post-hoc* test for multiple comparisons of all groups with the control group or two-way analysis of variance with Bonferroni *post-hoc* test for comparisons between untreated and paclitaxel-treated groups. The level of statistical significance was set at *P* ≤ 0.05. Analyses were performed using GraphPad Prism.

## Results

### Paclitaxel Promotes Secretion of Interleukin-1β From Macrophages Upon Costimulation With ATP

**Figure 1:**
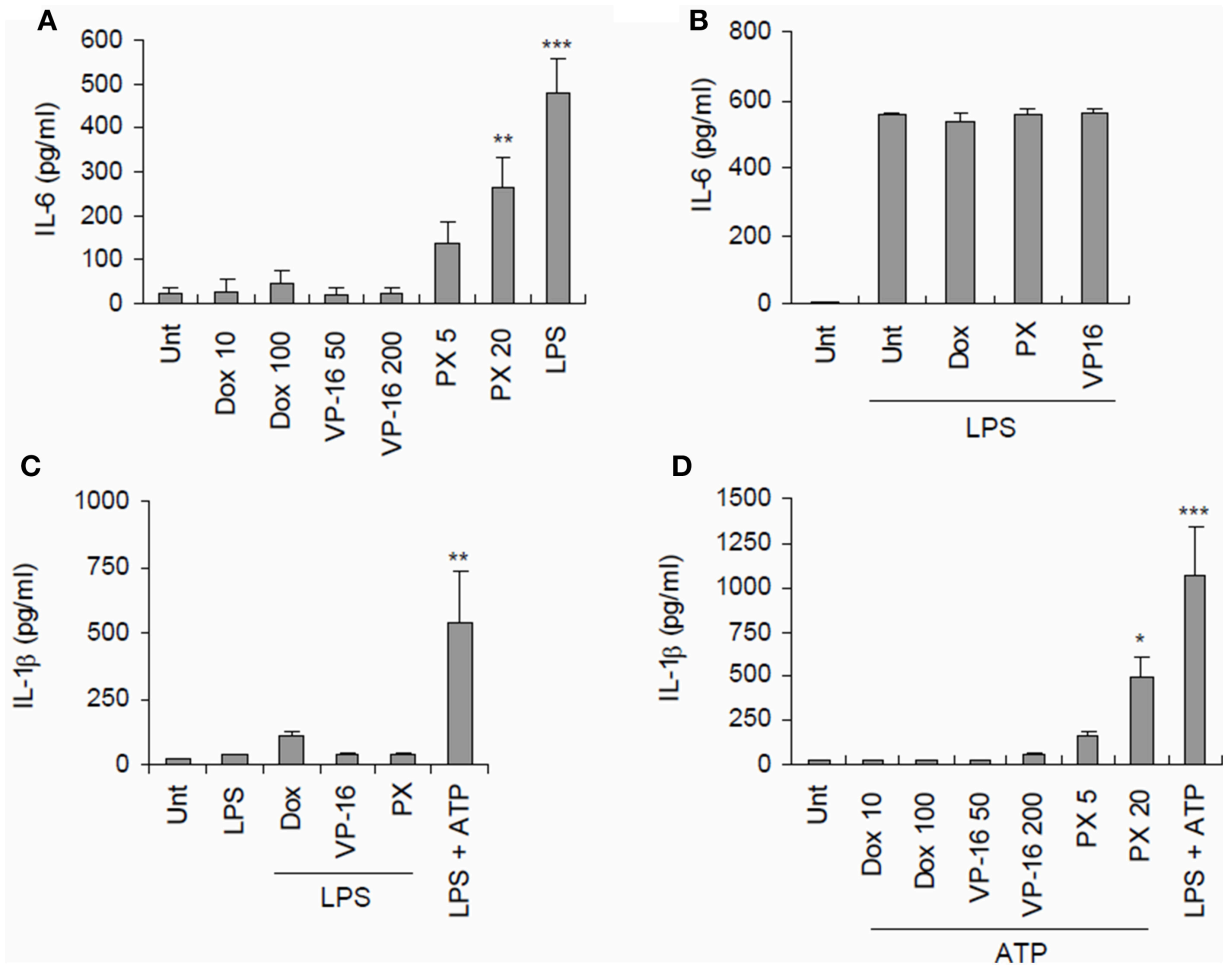
Paclitaxel treatment promotes secretion of proinflammatory cytokines from BMDMs. **(A)** Quantification of IL-6 in culture supernatants of mouse BMDMs untreated (Unt) or treated with doxorubicin (Dox, 10 or 100 μM), etoposide (VP-16, 50, or 200 μM), paclitaxel (PX, 5, or 20 μM) or LPS (0.25 μg/mL) for 3 h (*n* = 3). **(B)** Quantification of IL-6 in culture supernatants of mouse BMDMs primed with LPS (0.25 μg/mL) for 3 h, followed by treatment with doxorubicin (100 μM), paclitaxel (20 μM) or etoposide (200 μM) for 6 h (*n* = 2). **(C)** Quantification of IL-1β in culture supernatants of mouse BMDMs primed with LPS (0.25 μg/mL, 3 h), followed by treatment with doxorubicin (10 or 100 μM), etoposide (50 or 200 μM), and paclitaxel (5 or 20 μM) for 3 h or ATP (2.5 mM) for 30 min (*n* = 2–4). **(D)** Quantification of IL-1β in culture supernatants of mouse BMDMs treated with doxorubicin (10 or 100 μM), etoposide (50 or 200 μM), paclitaxel (5 or 20 μM) or LPS (0.25 μg/ml) for 3 h, followed by treatment with ATP (2.5 mM, 30 min) (*n* = 4). Data were expressed as the mean ± SEM. Asterisks indicate significant differences (^*^P < 0.05, ^**^P < 0.01, ^***^P < 0.001).

To first examine whether chemotherapeutic drugs could induce the secretion of pro-inflammatory cytokines from macrophages, three common anti-tumor drugs, doxorubicin, etoposide, and paclitaxel, were used to treat BMDMs. Among the tested drugs, only paclitaxel caused considerable production of IL-6 in BMDMs (Figure 1A). However, no chemotherapeutic drug further increased the LPS-triggered IL-6 production (Figure 1B). Subsequently, we examined whether these anti-tumor drugs could mediate the activation of inflammasome signaling, as measured by IL-1β secretion. With LPS priming, all the tested drugs failed to promote the significant secretion of IL-1β in BMDMs (Figure 1C). Meanwhile, with ATP costimulation, paclitaxel-, but not doxorubicin or etoposide induced robust secretion of IL-1β from BMDMs (Figure 1D). This finding raises a possibility that paclitaxel treatment can trigger inflammasome activation and IL-1β secretion from macrophages under certain conditions.

### Paclitaxel Promotes NLRP3-Dependent Caspase-1 Activation in the Presence of an NLRP3-Activating Second Signal

**Figure 2:**
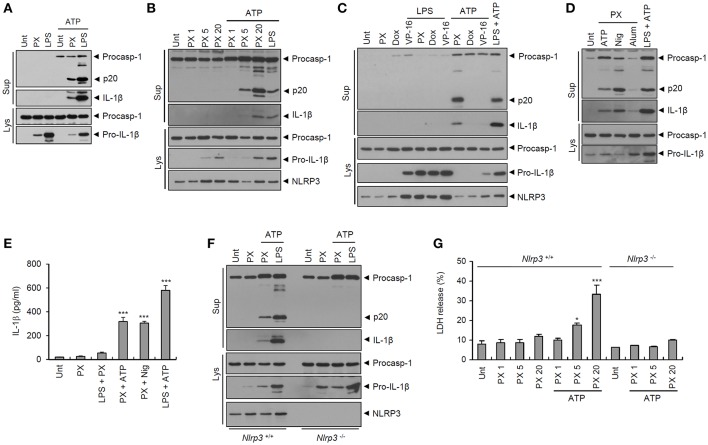
Paclitaxel treatment facilitates NLRP3-dependent caspase-1 activation in BMDMs. **(A)** Immunoblots from mouse BMDMs untreated (Unt) or treated with paclitaxel (PX, 5 μM) or LPS (0.25 μg/mL) for 3 h, followed by treatment with ATP (2.5 mM, 30 min), as indicated. **(B)** Immunoblots from mouse BMDMs untreated (Unt) or treated with paclitaxel (PX, 1~20 μM) or LPS (0.25 μg/mL) for 3 h, followed by treatment with ATP (2 mM, 30 min). **(C)** Immunoblots from mouse BMDMs untreated (Unt) or treated with paclitaxel (PX, 5 μM), doxorubicin (Dox, 10 μM) or etoposide (VP-16, 50 μM) alone for 6 h, or 3 h with LPS pretreatment (0.25 μg/mL, 3 h) or with ATP post-treatment (2.5 mM, 30 min), as well as with LPS, followed by ATP treatment. **(D)** Immunoblots from mouse BMDMs untreated (Unt) or treated with paclitaxel (PX, 5 μM) or LPS (0.25 μg/mL) for 3 h, followed by treatment with ATP (2 mM, 30 min), nigericin (Nig, 5 μM, 40 min), or alum (250 μg/mL, 7 h), as indicated. **(E)** Quantification of IL-1β in culture supernatants of mouse BMDMs treated with paclitaxel (PX, 5 μM, 3 h) in the presence of LPS pretreatment (0.5 μg/mL, 3 h), or treated with paclitaxel or LPS, followed by ATP (3 mM, 30 min) or nigericin (Nig, 5 μM, 40 min) (*n* = 3–4). **(F)** Immunoblots from *Nlrp3*^+/+^ or *Nlrp3*^−/−^ mouse BMDMs treated with paclitaxel (5 μM, 3 h), or LPS (0.25 μg/mL, 3 h), followed by ATP treatment (2 mM, 30 min). **(G)** LDH release into culture supernatants of *Nlrp3*^+/+^ (*n* = 3) or *Nlrp3*^−/−^ (*n* = 2) mouse BMDMs treated with paclitaxel (PX, 1, 5 or 20 μM, 3 h), followed by treatment with ATP (2 mM, 30 min). **(A–D,F)** Culture supernatants (Sup) or cell lysates (Lys) were immunoblotted with the indicated antibodies. **(E,G)** Asterisks indicate significant differences (^*^P < 0.05, ^***^P < 0.001).

Thereafter, we examined whether paclitaxel indeed drives caspase-1 activation in macrophages, as determined by the presence of active caspase-1 (p20) in culture supernatants. Consistent with the data shown in Figure 1D, paclitaxel treatment induced robust activation of caspase-1 and subsequent secretion of active IL-1β in BMDMs only with ATP costimulation (Figures 2A,B) but not following LPS priming (Figure 2C). Generally, ATP is considered a common second signal for NLRP3 inflammasome activation, which requires two independent signals, a priming signal 1 such as LPS and an activation signal 2 such as ATP (22). Therefore, we further tested whether paclitaxel can activate caspase-1 in the presence of another NLRP3 activation signal 2, such as nigericin or alum crystals, and demonstrated that costimulation with nigericin or alum after paclitaxel treatment also led to robust activation of caspase-1 and IL-1β (Figures 2D,E).

We then explored the NLRP3 dependency of paclitaxel-induced inflammasome activation. Paclitaxel plus ATP stimulation induced strong activation of caspase-1 and IL-1β in *Nlrp3*-expressing but not in *Nlrp3*-deficient BMDMs (Figure 2F). In addition, treatment with paclitaxel alone did not cause a significant cell death of BMDMs in our experimental settings, whereas paclitaxel treatment, followed by ATP stimulation, resulted in a considerable cell death (Figure 2G). This paclitaxel/ATP-mediated cell death was not observed in *Nlrp3*-deficient BMDMs (Figure 2G). These findings indicate that paclitaxel/ATP treatment is able to promote NLRP3 inflammasome activation and a subsequent caspase-1-dependent pyroptotic cell death of macrophages.

### Paclitaxel Treatment, Followed by ATP Costimulation, Promotes Assembly of the NLRP3 Inflammasome.

**Figure 3:**
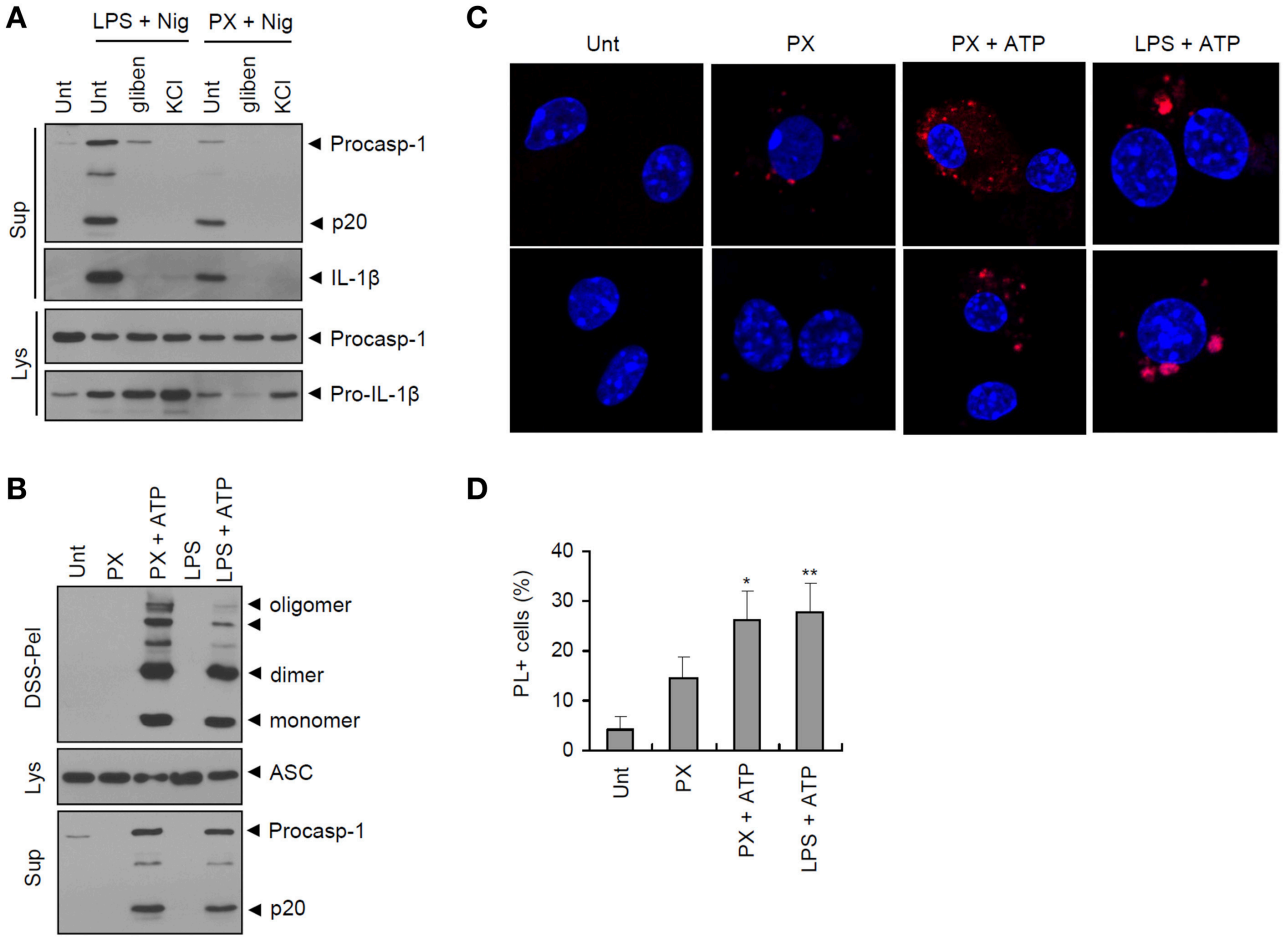
Paclitaxel treatment in the presence of an NLRP3 agonist promotes the assembly of the NLRP3 inflammasome. **(A)** Immunoblots from mouse BMDMs untreated (Unt) or primed with LPS (0.25 μg/mL, 3 h) or paclitaxel (5 μM, 3 h) in the presence of glibenclamide (100 μM) or KCl (30 mM), followed by treatment with nigericin (5 μM, 40 min). Culture supernatants (Sup) or cell lysates (Lys) were immunoblotted with the indicated antibodies. **(B)** Immunoblots of disuccinimidyl suberate (DSS)-crosslinked pellets (DSS-pel), cellular lysates (Lys) or cultural supernatants (Sup) from mouse BMDMs treated with LPS (0.25 μg/mL, 3 h) or paclitaxel (5 μM, 3 h), followed by treatment with ATP (3 mM, 30 min). **(C)** Proximity ligation (PL) assay of NLRP3 and ASC in mouse BMDMs treated with paclitaxel (PX, 5 μM, 3 h) or LPS (0.25 μg/mL, 3 h), followed by treatment with ATP (2.5 mM, 30 min). PL signals (red) represent the molecular association of NLRP3 and ASC. Data are shown as a representative image from five-independent samples. Scale bars, 10 μm. **(D)** Relative percentages of PL signal-positive cells (*n* = 5). Data are expressed as the mean ± SEM. Asterisks indicate significant differences (^*^P < 0.05, ^**^P < 0.01).

Next, we examined whether paclitaxel/ATP treatment indeed drives the assembly of the NLRP3 inflammasome. Potassium (K^+^) efflux is considered an essential upstream requirement for NLRP3 inflammasome assembly (23). Inhibition of K^+^ efflux by glibenclamide or extracellular KCl treatment completely blocked caspase-1 activation by paclitaxel/nigericin treatment (Figure 3A), indicating that K^+^ efflux was mediated by paclitaxel-induced inflammasome assembly. Consistently, paclitaxel/ATP treatment caused strong oligomerization of ASC, as determined by the DSS-mediated cross-linking assay (Figure 3B). In addition, paclitaxel treatment with ATP costimulation increased the protein-protein interaction between NLRP3 and ASC, as measured by PL assay (Figures 3C,D). These observations demonstrated that paclitaxel could facilitate the K^+^ efflux-dependent assembly of the NLRP3 inflammasome in macrophages, as shown by the interaction of NLRP3 with ASC and ASC oligomerization.

### Paclitaxel Mediates Transcriptional Induction of Proinflammatory Cytokines in a TLR4-Dependent Manner

**Figure 4:**
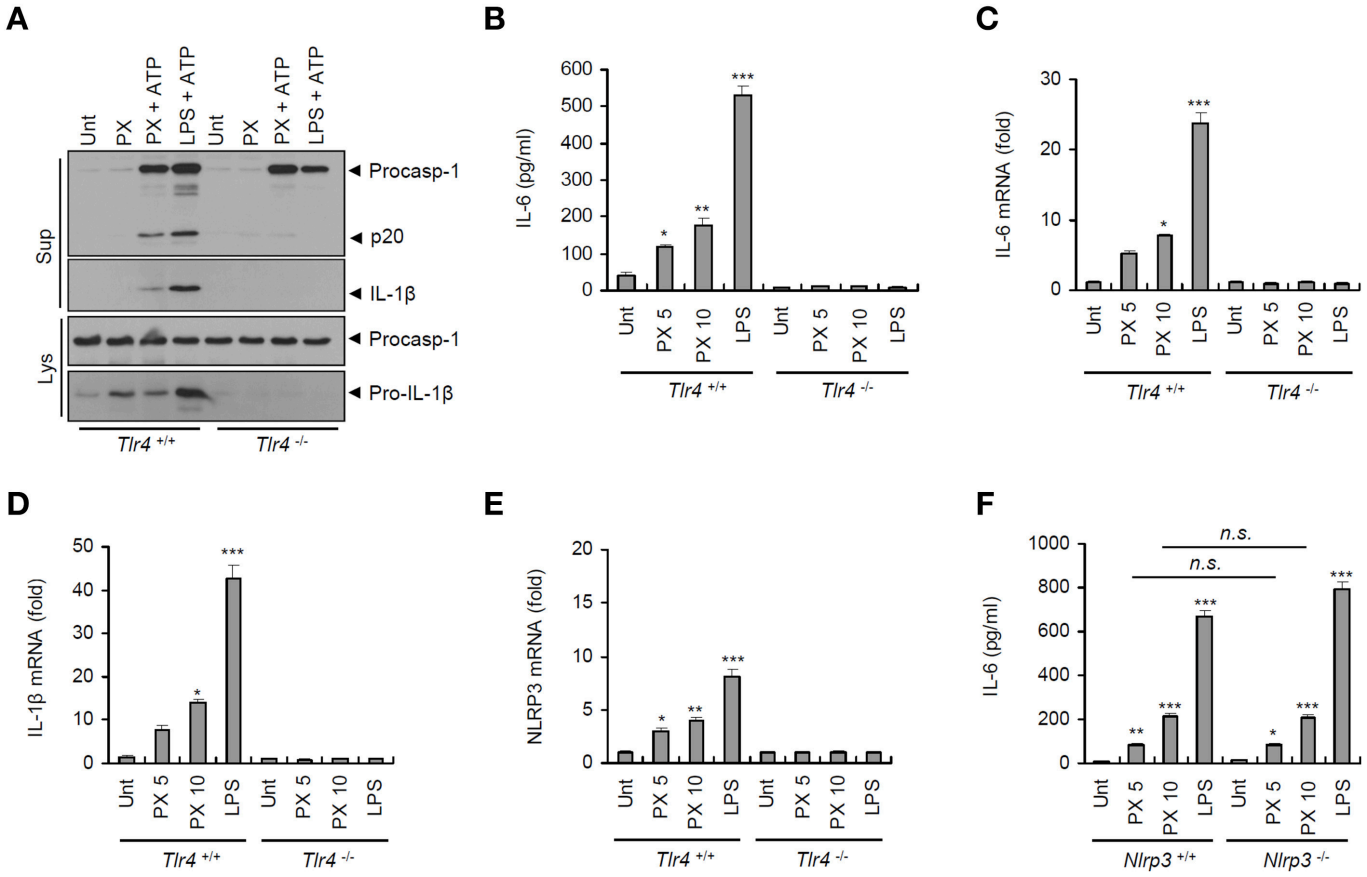
Paclitaxel treatment promotes TLR4-dependent inflammasome activation and proinflammatory cytokine production. **(A)** Immunoblots of cultural supernatants (Sup) or cellular lysates (Lys) from *Tlr4*^+/+^ or *Tlr4*^−/−^ mouse BMDMs treated with paclitaxel (PX, 5 μM, 3 h) or LPS (0.25 μg/mL, 3 h), followed by ATP treatment (3 mM, 30 min). **(B)** Quantification of IL-6 in culture supernatants of *Tlr4*^+/+^ or *Tlr4*^−/−^ mouse BMDMs treated with paclitaxel (5 or 10 μM, 3 h) or LPS (0.5 μg/mL, 3 h) (*n* = 3). **(C–E)** Quantification of *Il-6* **(C)**, *Il-1*β **(D)**, or *Nlrp3* **(E)** mRNA levels in *Tlr4*^+/+^ or *Tlr4*^−/−^ mouse BMDMs treated with paclitaxel (5 or 10 μM, 3 h) or LPS (0.5 μg/mL, 3 h) (*n* = 3–6). **(F)** Quantification of IL-6 in culture supernatants of *Nlrp3*^+/+^ or *Nlrp3*^−/−^ mouse BMDMs treated with paclitaxel (5 or 10 μM, 3 h) or LPS (0.5 μg/mL, 3 h) (*n* = 3). Data are expressed as the mean ± SEM. Asterisks indicate significant differences (^*^P < 0.05, ^**^P < 0.01, ^***^P < 0.001, *n.s*., not significant).

To understand how paclitaxel treatment drives activation of the NLRP3 inflammasome, we first examined the requirement for TLR4 signaling. Previous studies have reported that paclitaxel induced TLR4-mediated signaling in many cancer cells (7, 8). It was of particular interest that paclitaxel/ATP-induced caspase-1 activation and IL-1β secretion was completely abrogated in *Tlr4*-deficient BMDMs (Figure 4A). Similar to its effect on IL-1β secretion, paclitaxel treatment led to the secretion of IL-6 in wild-type BMDMs but not in *Tlr4*-deficient macrophages (Figure 4B). Indeed, paclitaxel induced a robust mRNA expression of *Il-6, Il-1*β, and *Nlrp3* in a TLR4-dependent manner (Figures 4C–E). However, paclitaxel-promoted production of IL-6 protein and mRNA was independent of NLRP3 presence (Figure 4F and Supplementary Figure 1). These results strongly support the hypothesis that paclitaxel can induce robust transcription of proinflammatory cytokines in a TLR4-dependent manner.

### Paclitaxel Mediates JNK-Implicating Non-transcriptional Priming of NLRP3 Inflammasome Activation

**Figure 5:**
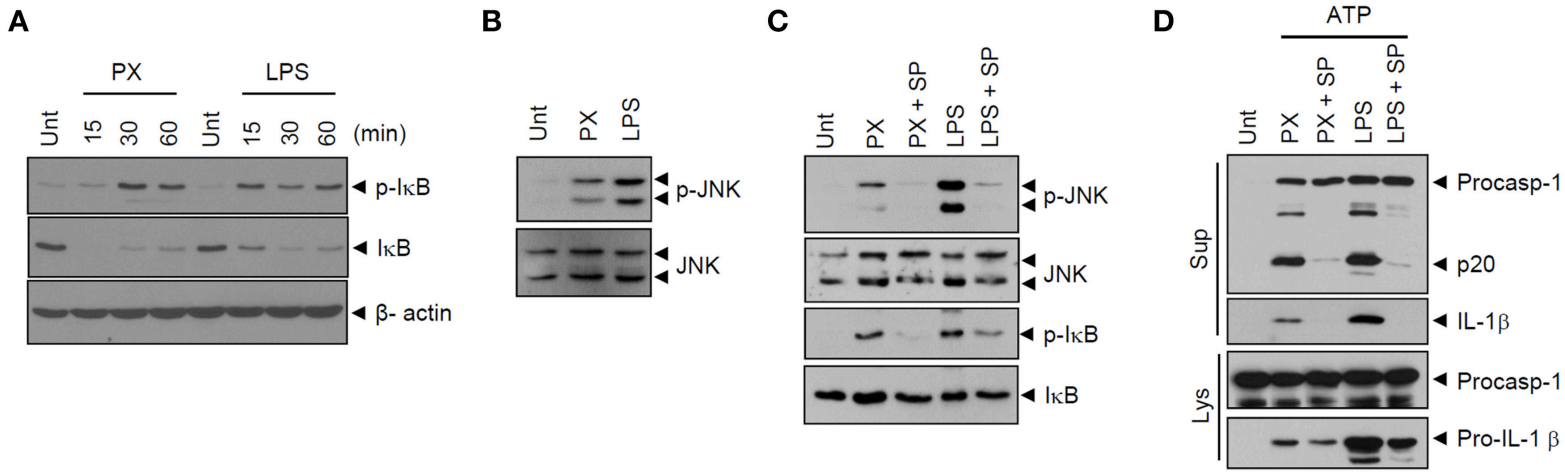
Paclitaxel mediates JNK signaling-dependent priming of NLRP3 inflammasome activation. **(A)** Immunoblots of cellular lysates from mouse BMDMs untreated (Unt) or treated with paclitaxel (5 μM) or LPS (0.25 μg/mL) for 15, 30, or 60 min. **(B)** Immunoblots of cellular lysates from mouse BMDMs treated with paclitaxel (5 μM) or LPS (0.5 μg/mL) for 3 h. **(C)** Immunoblots of cultural supernatants or cellular lysates from mouse BMDMs treated with paclitaxel (5 μM, 3 h) or LPS (0.25 μg/mL, 3 h) after pretreatment with SP600125 (20 μM, 30 min). **(D)** Immunoblots of cultural supernatants or cellular lysates from mouse BMDMs treated with paclitaxel (5 μM, 3 h) or LPS (0.25 μg/mL, 3 h) after pretreatment with SP600125 (20 μM, 30 min), followed by treatment with ATP (2.5 mM, 30 min).

We then examined TLR4 downstream signaling pathways, such as NF-κB and MAP kinase signaling, in macrophages upon paclitaxel stimulation. Similar to LPS treatment, paclitaxel caused robust phosphorylation and degradation of IκB (Figure 5A), indicating that paclitaxel treatment led to the activation of NF-κB pathways in macrophages. In addition, paclitaxel drove robust phosphorylation of JNK in BMDMs (Figure 5B). NLRP3 inflammasome activation requires non-transcriptional priming steps downstream of TLR (24–27). A recent study has suggested that TLR4-mediated activation of JNK signaling pathways is an essential non-transcriptional priming step for NLRP3 activation (28). Interestingly, the JNK-selective inhibitor SP600125 remarkably blocked paclitaxel-induced IκB phosphorylation in BMDMs (Figure 5C). Furthermore, SP600125 clearly abolished paclitaxel/ATP-triggered caspase-1 activation (Figure 5D). These data support the hypothesis that paclitaxel-mediated JNK signaling is potentially implicated in the non-transcriptional priming step of paclitaxel-mediated NLRP3 inflammasome activation.

**Figure 6:**
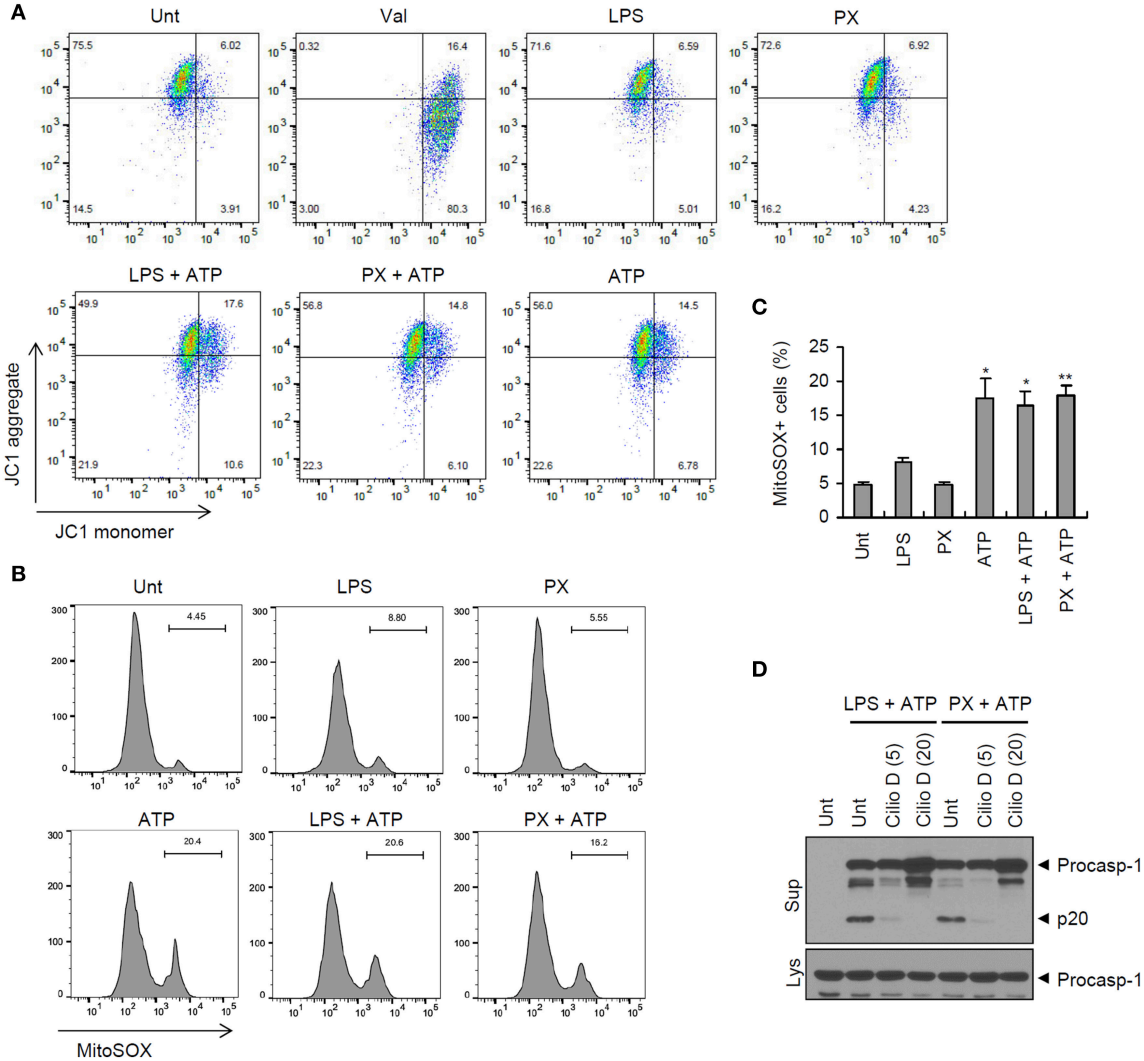
Paclitaxel does not promote mitochondrial damages. **(A,B)** Flow cytometric analysis of BMDMs treated with paclitaxel (5 μM, 3 h) or LPS (0.25 μg/mL, 3 h), followed by the treatment with ATP (2.5 mM, 30 min), or treated with valinomycin (Val, 5 μM, 30 min, A), after staining with JC-1 **(A)** or MitoSOX **(B)**. **(C)** Quantification of MitoSOX-positive cells in **(B)**. Asterisks indicate significant differences from untreated control (^*^P < 0.05, ^**^P < 0.01; *n* = 2, untreated and ATP; *n* = 3, others). **(D)** Immunoblots of culture supernatants or cellular lysates from mouse BMDMs treated with paclitaxel (5 μM, 3 h) or LPS (0.25 μg/mL, 3 h) after pretreatment with cilliobrevin D (5 or 20 μM, 30 min), followed by treatment with ATP (2.5 mM, 30 min).

Given that mitochondrial damage may contribute to the priming or activation of NLRP3 inflammasome (16, 29), we then examined whether paclitaxel treatment could induce mitochondrial impairments, by measuring the loss of the mitochondrial membrane potential and the production of mitochondrial ROS. The results showed that paclitaxel/ATP treatment resulted in a reduced mitochondrial membrane potential (75.5–56.8%), as demonstrated by JC-1 staining, but this reduction was due to ATP treatment rather that to treatment with paclitaxel alone (Figure 6A). Similarly, treatment with ATP alone led to an increase in mitochondrial ROS production, whereas paclitaxel treatment did not change the levels of mitochondrial ROS (Figures 6B,C and Supplementary Figure 2). These observations demonstrated that mitochondrial damages were mainly induced by NLRP3-activating second signals, such as ATP, but not by paclitaxel treatment. On the other hand, the inhibition of mitochondrial transport by ciliobrevin D markedly blocked paclitaxel/ATP-mediated caspase-1 activation (Figure 6D). Meanwhile, a recent our previous study has shown that mitochondrial transport is also driven by ATP treatment (30). These findings further support the hypothesis that paclitaxel does not function as an activation signal 2 for NLRP3 inflammasome activation.

### Paclitaxel-Induced Priming Triggers Interleukin-1β Secretion Upon Costimulation With Products Released From Damaged Cells

**Figure 7:**
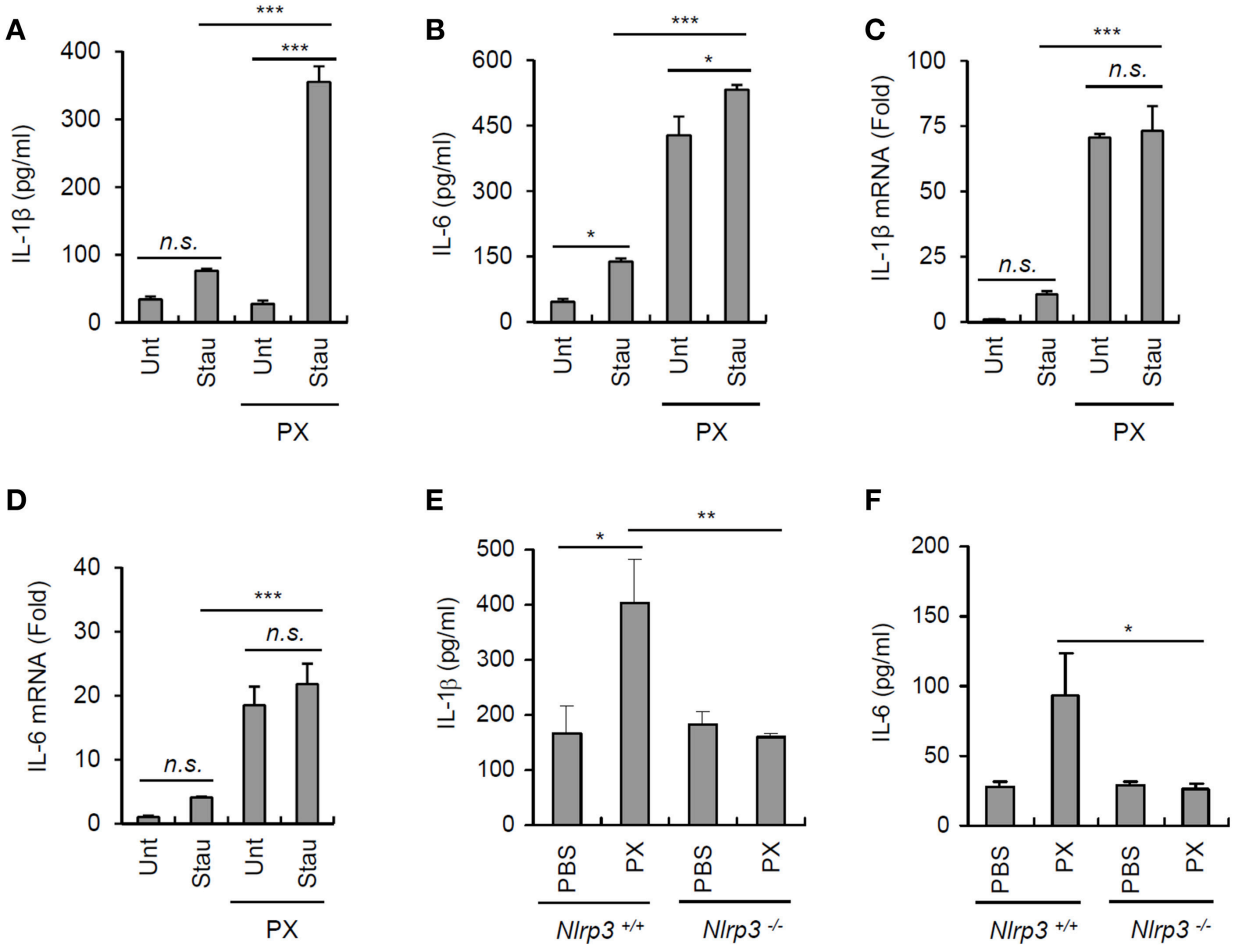
Paclitaxel promotes IL-1β secretion under physiological conditions. **(A,B)** Untreated or staurosporine (1 μg/ml, 24 h)-treated A549 cells were washed and incubated with a fresh medium for an additional 18 h to collect culture supernatants. Untreated or paclitaxel (5 μM, 3 h)-pretreated BMDMs were further incubated for 18 h with conditioned medium (A549 culture supernatants + fresh medium, 2:1), and IL-1β or IL-6 levels were assayed in the supernatants by ELISA (*n* = 3). **(C,D)** Quantification of IL-1β (C) or IL-6 (D) mRNA levels in BMDMs treated as in **(A,B)** (*n* = 3). **(E,F)** Quantification of IL-1β (D) or IL-6 (E) in extracts of the spleen from *Nlrp3*^+/+^ or *Nlrp3*^−/−^ mice administered the vehicle or paclitaxel (40 mg/kg) for 24 h (*n* = 3–5). Asterisks indicate significant differences (^*^P < 0.05, ^**^P < 0.01, ^***^P < 0.001). n.s., not significant.

Extracellular ATP is considered a main danger signal released from dead or injured cells (31). To examine whether culture supernatants from injured cells can facilitate the paclitaxel-primed inflammasome activation, A549 cells were treated with staurosporine to induce a robust cell death. Then, the cultural supernatants from staurosporine-treated A549 cells were collected and used to treat unprimed or paclitaxel-primed BMDMs. Similar levels of IL-1β were detected from culture supernatants of staurosporine-treated A549 cells, compared with that of untreated A549 cells (Figure 7A). On the contrast, there was a slight increase in IL-6 production from A549 cells by the treatment of staurosporine (Figure 7B). Intriguingly, supernatants of the staurosporine-treated cultures promoted robust IL-1β secretion by paclitaxel-primed BMDMs but not by unprimed BMDMs (Figure 7A). However, culture supernatants from staurosporine-treated A549 cells alone failed to induce the production of IL-1β and IL-6 mRNA in BMDMs (Figures 7C,D). These data indicate that paclitaxel provides a priming signal, and products released from damaged cells, possibly containing ATP, can function as an activation signal for the NLRP3 inflammsome activation in our experimental settings.

To test whether paclitaxel treatment can induce inflammasome activation *in vivo*, mice were intraperitoneally challenged with paclitaxel. Consequently, paclitaxel-treated wild-type mice showed a marked increase in the level of IL-1β in the spleen, whereas *Nlrp3*-deficient mice did not show any changes in the IL-1β levels (Figure 7E). Paclitaxel administration also increased the IL-6 levels in the spleen of wild-type mice but not of *Nlrp3*^−/−^ mice (Figure 7F). All the findings strongly demonstrate that the administration of the chemotherapeutic drug paclitaxel is able to promote the priming and activation of NLRP3 inflammasome signaling, which may affect the efficacy of paclitaxel, depending on the adjacent conditions.

## Discussion

Many chemotherapeutic agents have been previously shown to induce the production of proinflammatory cytokines in cancer or myeloid cells (32). In our experimental settings, the chemotherapy drugs doxorubicin and etoposide did not trigger the IL-6 production, while paclitaxel induced a robust expression of IL-6 in TLR4-expressing macrophages. Given that paclitaxel can act as a direct ligand for TLR4, this drug is more likely to promote transcriptional production of proinflammatory cytokines than other anticancer drugs are.

Some previous studies have also shown that chemotherapeutic drugs caused secretion of IL-1β by LPS-primed macrophages (33, 34). However, all three tested drugs failed to induce robust IL-1β production and caspase-1 activation in BMDMs in combination with LPS priming in our study. However, paclitaxel treatment, followed by a NLRP3-activating second signal such as ATP or nigericin, markedly induced caspase-1 activation and IL-1β secretion in BMDMs. These findings demonstrate that paclitaxel can provide a priming signal for NLRP3 inflammasome activation.

As a further support of this priming effect of paclitaxel, we found that paclitaxel treatment led to phosphorylation of IκB and JNK, confirming that paclitaxel mediates the activation of TLR4 downstream signaling pathways. The activation of IκB/NF-κB signaling is responsible for the induction of transcription of proinflammatory cytokines by paclitaxel (35). However, our data clearly indicate that paclitaxel can trigger NLRP3-dependent caspase-1 activation, which does not require transcription of proinflammatory cytokines. Therefore, we focused on JNK activation by paclitaxel treatment. Indeed, the JNK-selective inhibitor SP600125 clearly abrogated paclitaxel/ATP-induced caspase-1 activation. These data demonstrate that paclitaxel-induced non-transcriptional priming is required for activation of the NLRP3 inflammasome, although the molecular mechanism of paclitaxel-induced priming still remains to be elucidated.

Interestingly, cell-free medium from staurosporine-treated injured cells, probably containing danger-associated molecular patterns (DAMPs), failed to induce robust production of IL-1β or IL-6 in BMDMs. However, these DAMP-containing supernatants induced marked IL-1β secretion from paclitaxel-primed BMDMs. This result further supports the hypothesis that paclitaxel can trigger NLRP3 inflammasome activation in the environment adjacent to dead or injured cells. Administration of paclitaxel to mice also led to a robust increase in IL-1β levels in the spleens of *Nlrp3*-expressing but not *Nlrp3*-deficient mice. These observations indicate that treatment with paclitaxel alone is able to promote NLRP3 inflammasome activation in a certain physiological environment.

It remains unclear whether paclitaxel-induced inflammasome activation can affect the chemotherapeutic potential of the drugs. Many previous studies have suggested that proinflammatory cytokines, including IL-1β, may attenuate the efficacy of chemotherapeutic drugs, including paclitaxel (32, 36). This hypothesis is supported by the observation that treatment with an IL-1 receptor antagonist improved the antitumor effects of chemotherapy drugs (13, 15). In this context, we can assume that paclitaxel-mediated inflammasome activation might attenuate its chemotherapeutic potential. On the other hand, a recent study has shown that paclitaxel increases the antitumor immunity via reprogramming of M2-like macrophages into cells with an M1-like phenotype, which enhances the antitumor potential of paclitaxel (10). Furthermore, the role of NLRP3 inflammasome in cancer development and progression remains controversial and conflicting. Depending on the tumor types and tissues, NLRP3 inflammasome could have either protective or aggravating effects on tumor growth or metastasis. Therefore, the effect of paclitaxel-mediated inflammasome activation needs to be further evaluated from a perspective of cancer therapy.

## Data Availability

All datasets generated for this study are included in the manuscript and/or the Supplementary Files.

## Ethics Statement

Protocols for the animal experiments (2017-0221) were approved by the Institutional Ethical Committee, Yonsei University College of Medicine. All experiments were performed in accordance with the approved guidelines of the Institutional Ethical Committee.

## Author Contributions

SS designed and performed most of the experiments. D-WS and IH performed the experiments. J-HP provided critical materials and scientific advice. SS and J-WY supervised the entire project and wrote the manuscript.

### Conflict of Interest Statement

The authors declare that the research was conducted in the absence of any commercial or financial relationships that could be construed as a potential conflict of interest.
