# Supplementary material for: Chemotherapeutic Agent Paclitaxel Mediates Priming of NLRP3 Inflammasome Activation
Source: Front Immunol. 2019 May 16;10:1108. doi: 10.3389/fimmu.2019.01108 (PMC6532018; doi:10.3389/fimmu.2019.01108)
Supplement: Supplementary file 1 [file Data_Sheet_1.PDF]

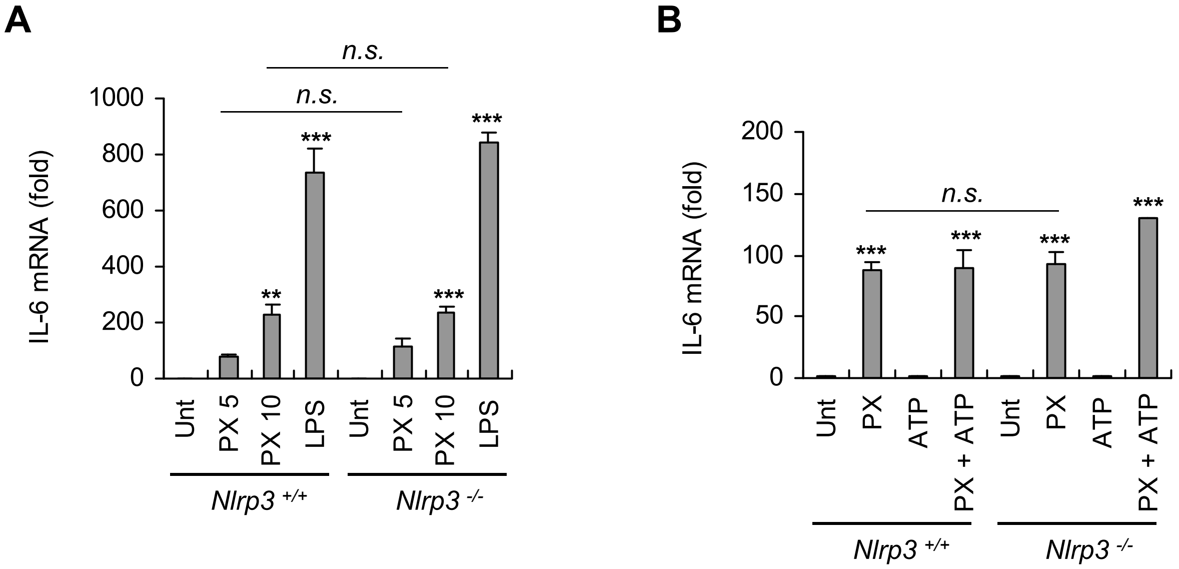

Supplementary Figure 1. NLRP3-independent production of IL-6 by paclitaxel treatment. (**A,B**) Quantification of *Il-6* mRNA levels in *Nlrp3* $^{+/+}$ or *Nlrp3* $^{-/-}$ mouse BMDMs treated with paclitaxel (5 or 10 μM, A; 5 μM, B) or LPS (0.25 μg/mL) for 3 h, followed by ATP treatment (2.5 mM, 30 min, B). (*n* = 3) Asterisks indicate significant differences. (**P<0.01, ***P<0.001) *n.s.* not significant.

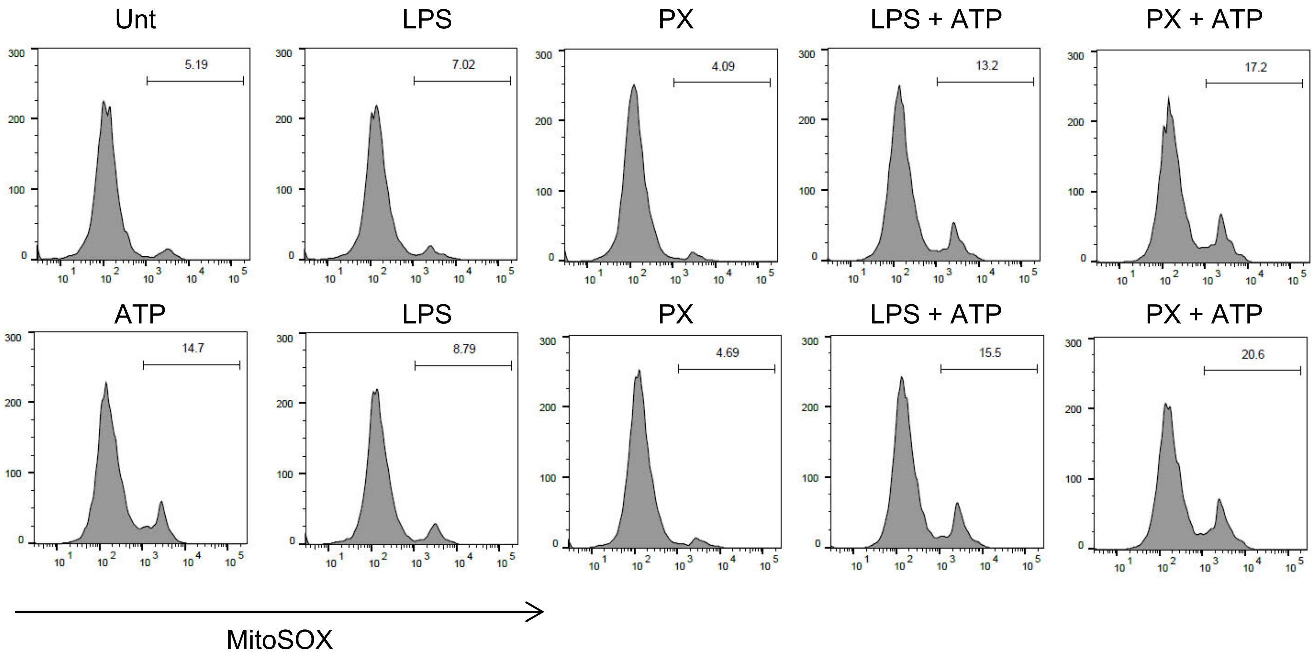

Supplementary Figure 2. Determination of mitochondrial ROS production in BMDMs. Flow cytometric analysis of BMDMs treated with paclitaxel (5 μM, 3 h) or LPS (0.25 μg/mL, 3 h), followed by the treatment with ATP (2.5 mM, 30 min), after staining with MitoSOX.
